# Supplementary material for: Sources of variation in the serum metabolome of female participants of the HUNT2 study
Source: Commun Biol. 2024 Nov 6;7:1450. doi: 10.1038/s42003-024-07137-x (PMC11541904; doi:10.1038/s42003-024-07137-x)
Supplement: Supplementary file 1 — Supplementary Information [file 42003_2024_7137_MOESM1_ESM.pdf]

Supplementary Information

Sources of variation in the serum metabolome of female participants of the HUNT2 study

Julia Debik[1*], Katarzyna Mrowiec[2*], Agata Kurczyk[3], Piotr Widłak[4], Karol Jelonek[2], Tone F. Bathen[5,6], Guro F. Giskeødegård[1,7]

[1]*Department of Public Health and Nursing, Norwegian University of Science and Technology, Trondheim, Norway.*

[2]*Center for Translational Research and Molecular Biology of Cancer, Maria Skłodowska-Curie National Research Institute of Oncology, Gliwice Branch, Gliwice, Poland.*

[3]*Department of Biostatistics and Bioinformatics, Maria Skłodowska-Curie National Research Institute of Oncology, Gliwice Branch, Gliwice, Poland.*

[4]*2nd Radiology Department, Medical University of Gdańsk, Gdańsk, Poland.*

[5]*Department of Circulation and Medical Imaging, Norwegian University of Science and Technology, Trondheim, Norway.*

[6]*Department of Radiology and Nuclear Medicine, St. Olav's University Hospital, Trondheim, Norway.*

[7]*Clinic of Surgery, St. Olav's University Hospital, Trondheim, Norway*

**Shared first author*

Corresponding authors:

Julia Debik (e-mail: julia.b.debik@ntnu.no)

Guro F. Giskeødegård (e-mail: guro.giskeodegard@ntnu.no)

TABLE OF CONTENTS

Figure S1. Proportion of missing lifestyle-related variables for the full study cohort

Figure S2. Principal component analysis (PCA) biplot plot for the lifestyle-defined clusters of participants and correlation plot showing correlations between principal components and lifestyle-variables.

Figure S3. Nuclear magnetic resonance (NMR)-defined clusters vs. lifestyle-defined clusters.

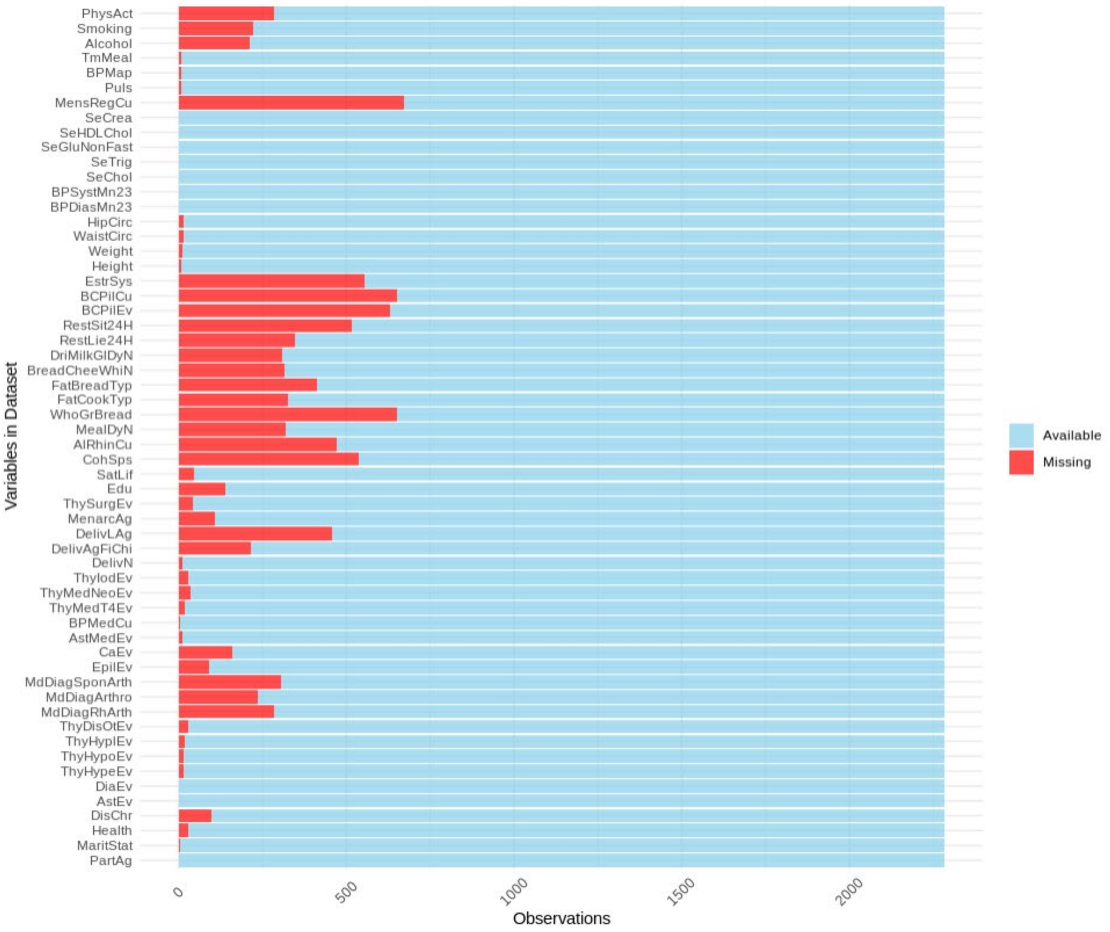

Figure S1 *Proportion of missing lifestyle-related variables for the full study cohort. Missing values have been imputed through the use of random forests.*

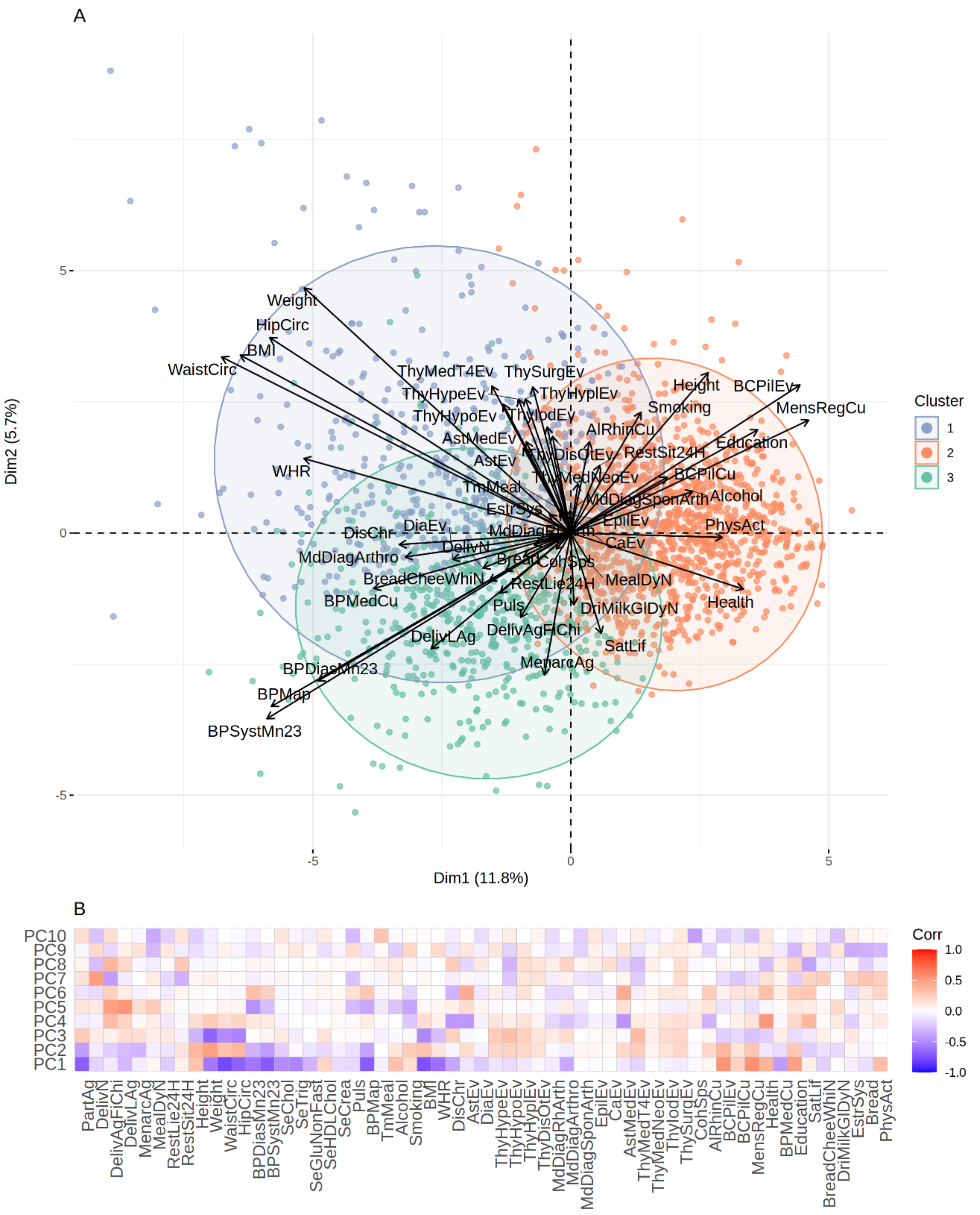

Figure S2 *PCA biplot for the lifestyle-defined clusters of participants (A). Correlations between the first ten principal components (PCs) and numeric and ordinal categorical lifestyle variables (B). PCA: Principal component analysis.*

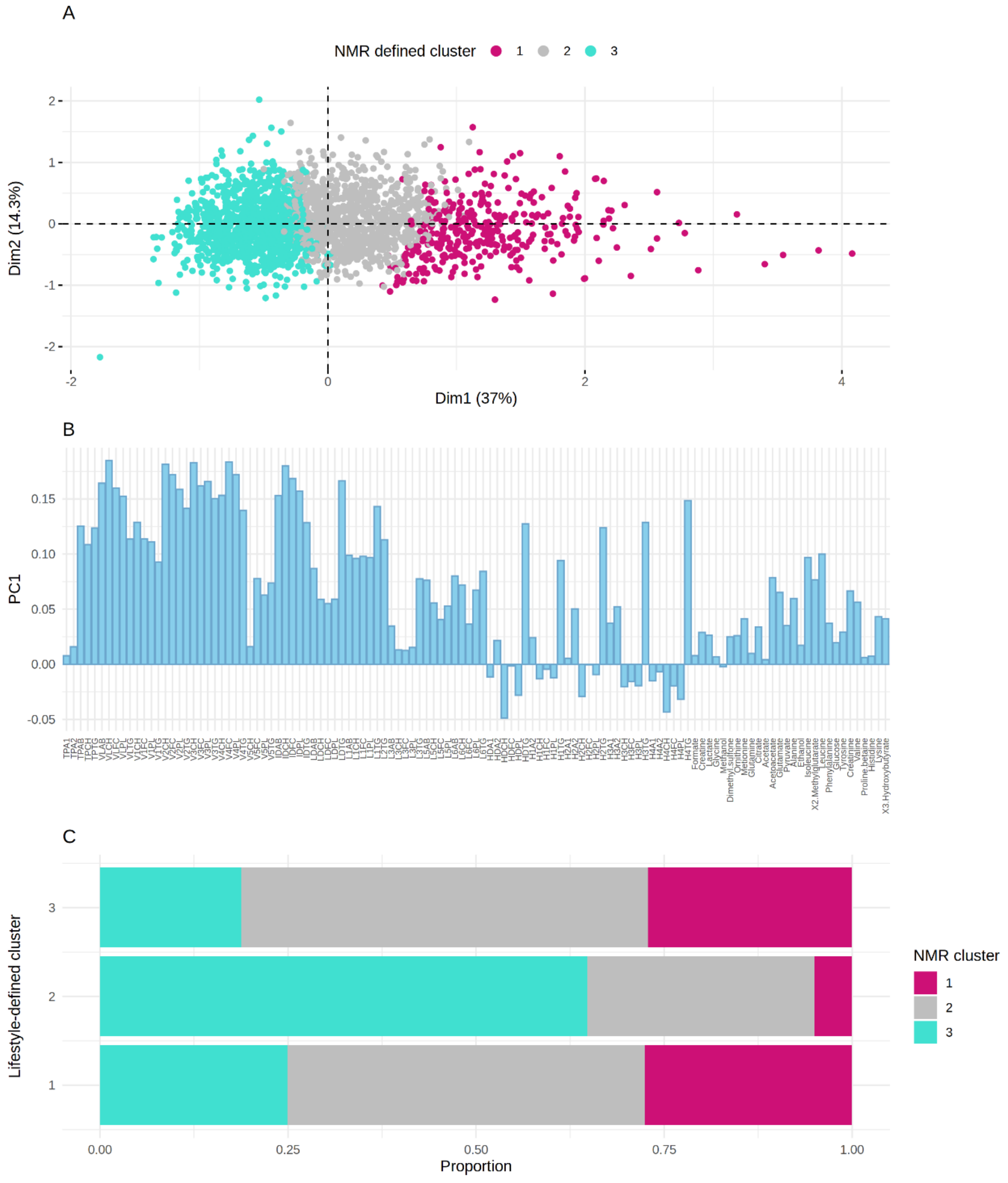

Figure S3 *NMR-defined clusters vs. lifestyle-defined clusters. Study participants clustered according to NMR-measured variables (lipoproteins and metabolites) projected onto a PCA scores plot (A). Principal component 1 (B). Proportion of NMR defined clusters in each lifestyle-defined cluster (C). NMR: Nuclear magnetic resonance; PCA: Principal component analysis*
