# Supplementary material for: Sources of variation in the serum metabolome of female participants of the HUNT2 study
Source: Commun Biol. 2024 Nov 6;7:1450. doi: 10.1038/s42003-024-07137-x (PMC11541904; doi:10.1038/s42003-024-07137-x)
Supplement: Supplementary file 2 — Description of Additional Supplementary Materials [file 42003_2024_7137_MOESM2_ESM.pdf]

Description of Additional Supplementary Files

File name: Supplementary Data 1

Description: Extended Table 1. Baseline characteristics of the study population. This table presents a selection of the available lifestyle-related variables (the variables showing the biggest differences across lifestyle-defined clusters) available for the study cohort and their distributions.

File name: Supplementary Data 2

Description: Variance explained (RSQ) values of metabolites by lifestyle related variables over the 10-fold cross validation from Ridge regression for lipoproteins, NMR-measured metabolites, MS-measured lipids and MS-measured metabolites.

File name: Supplementary Data 3

Description: Spearman correlations between lifestyle-related variables and NMR-measured lipoprotein subfractions, NMR-measured metabolites, aggregated levels of MS-measured lipids and MS-measured metabolites.

File name: Supplementary Data 4

Description: Mean relative differences (%) in concentrations of NMR-measured serum lipoproteins and metabolites, and MS-measured metabolites and lipids among participants of lifestyle-defined clusters 1 and 3 compared to participants in cluster 2.
