# Supplementary material for: Sources of variation in the serum metabolome of female participants of the HUNT2 study
Source: Commun Biol. 2024 Nov 6;7:1450. doi: 10.1038/s42003-024-07137-x (PMC11541904; doi:10.1038/s42003-024-07137-x)
Supplement: Supplementary file 7 — Reporting Summary [file 42003_2024_7137_MOESM7_ESM.pdf]

nature portfolio

Corresponding author(s): Julia Debik

Last updated by author(s): Aug 30, 2024

Reporting Summary

Nature Portfolio wishes to improve the reproducibility of the work that we publish. This form provides structure for consistency and transparency in reporting. For further information on Nature Portfolio policies, see our Editorial Policies and the Editorial Policy Checklist.

Statistics

For all statistical analyses, confirm that the following items are present in the figure legend, table legend, main text, or Methods section.

n/a	Confirmed	
☐	☒	The exact sample size (n) for each experimental group/condition, given as a discrete number and unit of measurement
☐	☒	A statement on whether measurements were taken from distinct samples or whether the same sample was measured repeatedly
☐	☒	The statistical test(s) used AND whether they are one- or two-sided<br>*Only common tests should be described solely by name; describe more complex techniques in the Methods section.*
☐	☒	A description of all covariates tested
☐	☒	A description of any assumptions or corrections, such as tests of normality and adjustment for multiple comparisons
☐	☒	A full description of the statistical parameters including central tendency (e.g. means) or other basic estimates (e.g. regression coefficient) AND variation (e.g. standard deviation) or associated estimates of uncertainty (e.g. confidence intervals)
☒	☐	For null hypothesis testing, the test statistic (e.g. F, t, r) with confidence intervals, effect sizes, degrees of freedom and P value noted<br>*Give P values as exact values whenever suitable.*
☒	☐	For Bayesian analysis, information on the choice of priors and Markov chain Monte Carlo settings
☒	☐	For hierarchical and complex designs, identification of the appropriate level for tests and full reporting of outcomes
☒	☐	Estimates of effect sizes (e.g. Cohen's d, Pearson's r), indicating how they were calculated

Our web collection on statistics for biologists contains articles on many of the points above.

Software and code

Policy information about availability of computer code

Data collection	Data has been collected from the HUNT biobank and from metabolic profiling through NMR spectroscopy.
Data analysis	Only easily available functions in the programming language R have been used for data analysis and no specialized algorithms have been developed. The functions and R-packages used have been specified in the manuscript.

For manuscripts utilizing custom algorithms or software that are central to the research but not yet described in published literature, software must be made available to editors and reviewers. We strongly encourage code deposition in a community repository (e.g. GitHub). See the Nature Portfolio guidelines for submitting code & software for further information.

Data

Policy information about availability of data

All manuscripts must include a data availability statement. This statement should provide the following information, where applicable:

- Accession codes, unique identifiers, or web links for publicly available datasets
- A description of any restrictions on data availability
- For clinical datasets or third party data, please ensure that the statement adheres to our policy

The Trøndelag Health Study (HUNT) has invited persons aged 13–100 years to four surveys between 1984 and 2019. Comprehensive data from more than 140,000 persons having participated at least once and biological material from 78,000 persons are collected. The data are stored in HUNT databank and biological material in HUNT biobank. HUNT Research Centre has permission from the Norwegian Data Inspectorate to store and handle these data. The key identification in the database

March 2021

is the personal identification number given to all Norwegians at birth or immigration, whilst de-identified data are sent to researchers upon approval of a research protocol by the Regional Ethical Committee and HUNT Research Centre. To protect participants' privacy, HUNT Research Centre aims to limit the storage of data outside HUNT databank, and cannot deposit data in open repositories. HUNT databank has precise information on all data exported to different projects and are able to reproduce these on request. There are no restrictions regarding data export given approval of applications to HUNT Research Centre. For more information, see http://www.ntnu.edu/hunt/data.

Human research participants

Policy information about studies involving human research participants and Sex and Gender in Research.

Reporting on sex and gender	Sex has been used consistently throughout the study as this is a biological trait in the context of our research.
Population characteristics	Population characteristics are presented in Table 1 of the manuscript.
Recruitment	Participants were recruited directly from the biobank. All individuals living in the North-Trøndelag aged 13 years upwards were invited to participate, and all individuals aged 18 years upwards were invited to donate a blood sample. Around 70 000 individuals participated in HUNT2, giving a participation rate of 66.7% for men and 75.5% for women. In the current study only women are included, thus the selection bias is quite limited compared to other large population biobanks, such as the UK biobank.
Ethics oversight	The study was approved by the Regional Ethical Committee. All HUNT participants have completed a written informed consent form, and the study was approved by the Ethics Committee of Central Norway (REK numbers #1995/8395 and #2017/2231). All ethical regulations relevant to human research participants were followed.

Note that full information on the approval of the study protocol must also be provided in the manuscript.

Field-specific reporting

Please select the one below that is the best fit for your research. If you are not sure, read the appropriate sections before making your selection.

☒ Life sciences ☐ Behavioural & social sciences ☐ Ecological, evolutionary & environmental sciences

For a reference copy of the document with all sections, see nature.com/documents/nr-reporting-summary-flat.pdf

Life sciences study design

All studies must disclose on these points even when the disclosure is negative.

Sample size	Sample size calculation was not relevant for this study
Data exclusions	Data were excluded based on missing data patterns as described in the manuscript
Replication	The reproducibility of metabolite measurements have been evaluated through coefficients of variation (CVs) on quality control samples from pooled serum samples of the participants which were run in parallel with serum samples during metabolomics profiling
Randomization	Metabolic profiling was done on the samples in a completely randomized order
Blinding	Blinding is not relevant for this manuscript

Reporting for specific materials, systems and methods

We require information from authors about some types of materials, experimental systems and methods used in many studies. Here, indicate whether each material, system or method listed is relevant to your study. If you are not sure if a list item applies to your research, read the appropriate section before selecting a response.

Materials & experimental systems

n/a	Involved in the study	
☒	☐	Antibodies
☒	☐	Eukaryotic cell lines
☒	☐	Palaeontology and archaeology
☒	☐	Animals and other organisms
☒	☐	Clinical data
☒	☐	Dual use research of concern

Methods

n/a	Involved in the study	
☒	☐	ChIP-seq
☒	☐	Flow cytometry
☒	☐	MRI-based neuroimaging
